# Atomistic Simulations of Mechanical Properties of Lignin

**DOI:** 10.3390/polym16243552

**Published:** 2024-12-19

**Authors:** Siteng Zhang, Yishayah Bension, Michael Shimizu, Ting Ge

**Affiliations:** Department of Chemistry and Biochemistry, University of South Carolina, Columbia, SC 29208, USA; siteng@email.sc.edu (S.Z.); yishayah@gmail.com (Y.B.); michael.shimizu@stonybrook.edu (M.S.)

**Keywords:** lignin, atomistic simulations, mechanical properties

## Abstract

The mechanical properties of lignin, an aromatic heteropolymer constituting 20–30% plant biomass, are important to the fabrication and processing of lignin-based sustainable polymeric materials. In this study, atomistic simulations are performed to provide microscopic insights into the mechanics of lignin. Representative samples of miscanthus, spruce, and birch lignin are studied. At room temperature below the glass transition temperature, the stress–strain curves for uniaxial compression and tensile loading are calculated and analyzed. The results show that lignin possesses rigidity with a Young’s modulus in the order of GPa and exhibits strain hardening under strong compression. Meanwhile, lignin is brittle and fails through the microscopic mechanism of cavitation and chain pullout under local tensile loading. In addition to the three common lignin samples, minimalist model systems of monodisperse linear chains consisting of only guaiacyl units and β-O-4 linkages are simulated. Systematic variation of the model lignin chain length allows a focused examination of the molecular weight effects. The results show that the molecular weight does not affect the Young’s modulus much, but higher molecular weight results in stronger strain hardening under compression. In the range of molecular weight studied, the lignin chains are not long enough to arrest the catastrophic chain pullout, explaining the brittleness of real lignin samples. This work demonstrates that the recently modified CHARMM force fields and the accompanying structural information of real lignin samples properly capture the mechanics of lignin, offering an in silico microscope to explore the atomistic details necessary for the valorizaiton of lignin.

## 1. Introduction

Lignin constitutes 20–30% of the biomass of plants and exists in plant cell walls together with cellulose and hemicellulose. Lignin is essentially an aromatic polymer with a heterogeneous chemical composition and a non-linear architecture, and it serves as the second-largest source of aromatic polymers on earth. While lignin is renewable, environmentally friendly, and abundant, and holds the potential for replacing conventional petroleum-derived polymers, more than 80% of natural lignin is disposed of as waste, or used as fuel. Creating lignin-based polymeric materials is at the frontier of sustainable polymer science and engineering [1]. Value-added applications of lignin include lignin-based adhesives [2], coatings [3], polymer composites [4,5,6,7], hydrogels [8], 3D-printing ink [9], and carbon fiber [10]. Critical to the fabrication and processing of lignin-based polymeric materials are the mechanical properties of lignin. In nature, the mechanical integrity of plant cells is maintained primarily by cellulose fibers, with strong longitudinal mechanical strength, but lignin acts as an indispensable space filler and collaborates with hemicellulose to bind cellulose fibers together for transverse mechanical strength. In value-added applications, lignin is blended with commercial polymers to enhance the mechanical properties of the composite and is also used as the rigid and macromolecular backbone of lignin-based thermoplastic elastomers [4,5]. Despite empirical knowledge of the mechanical properties of lignin, there is a knowledge gap regarding the structure–property relationship, and this prevents the rational design of the mechanics of lignin-based sustainable materials. In this article, we use atomistic simulations to examine the relationships between the mechanical properties of lignin samples and their molecular features.

## 2. Methods

Atomistic simulations are performed here to investigate lignin mechanics. The simulations have direct access to detailed microscopic information, which is hard to obtain in experiments, and therefore, can provide unparalleled microscopic insights into the structure–property relationship. Atomistic simulations of the mechanics of plant cell walls, including cellulose, hemicellulose, and lignin, have been performed recently to investigate the mechanical strength of bamboo fibrils [11] and the hygromechanical mechanisms of wood cell walls [12]. Atomistic simulations have also been performed to study the effects of heat and moisture on the elastic modulus of model lignin molecules [13]. However, atomistic simulations on the mechanics of lignin molecules with molecular weights, chemical compositions, and molecular architectures close to experimental samples have not been performed systematically. Furthermore, existing atomistic simulations have focused on the elastic response of lignin, and therefore, simulation work has yet to be extended to cover the plastic mechanics of lignin upon large deformation. Comprehensive simulations of lignin mechanics with rich microscopic features and extended mechanical response regimes are necessary for the interdisciplinary engineering of lignin-based polymeric materials and also distinguish this computational research from previous studies in the field.

**Figure 1:**
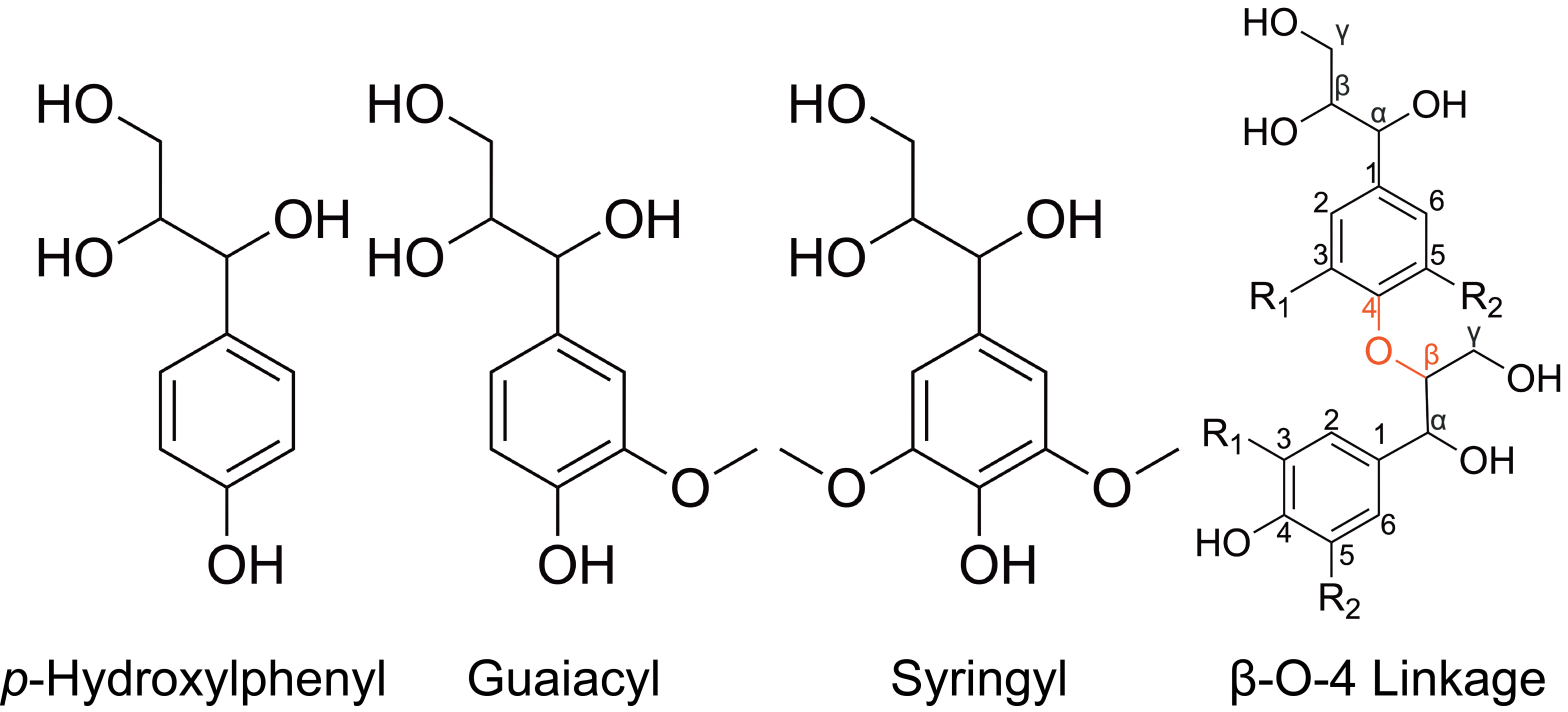
Chemical structures of three most common lignin monomers, H, G, and S, and the most common lignin linkage, β-O-4.

Atomistic simulations of lignin have just emerged, partly because the technical difficulty with modeling the complex structure of a lignin molecule has been addressed only recently. Unlike the well-defined structure of cellulose and hemicellulose, the molecular structure of lignin is described statistically by breaking down a lignin molecule into its constituent basic units, the *p*-Hydroxylphenyl (H), guaiacyl (G), and Syringyl (S) lignin monomers, and various linkages between the monomers. For instance, the G monomer and the β-O-4 linkage between two G monomers are the most common lignin monomer and linkage, respectively. The chemical structures of H monomer, G monomer, S monomer, and the β-O-4 linkage between two monomers are shown in Figure 1. Early atomistic simulations approximated lignin molecules as linear polymers consisting of G monomers and β-O-4 linkages only. In 2018, LigninBuilder was published by Vermaas et al. [14] and it has greatly facilitated the building of lignin molecules with well-controlled molecular weights, chemical compositions in terms of the ratios of various monomers and linkages, and molecular architectures in terms of the branching coefficient. The molecular features of the atomistic simulation samples created by LigninBuilder closely match those revealed by the analytical chemistry of natural lignin samples. Moreover, these samples have been used to study the conformations and solubility of lignin in different solvents, showing consistency with experimental observations [15]. The builder allows one to focus on the relation of mechanical properties to lignin’s structure in the simulations rather than the characterization and reproduction of lignin’s structure.

Three representative lignin samples were simulated with the structural information provided by LigninBuilder [14]. The three samples were the lignin of miscanthus, birch, and spruce, which represent grass, hardwood, and softwood, respectively. As shown in Table 1, each sample has a distribution of molecular weights, a unique chemical composition in terms of the fractions of the H, G, and S monomers, and a different branching coefficient that quantifies the fraction of monomers at branching points. The chain number of all three simulation samples is M=800. Additionally, model lignin samples were produced to further study the molecular weight effects. Each model lignin sample consists of linear chains made of G monomers and β-O-4 linkages (poly-G). As such, the chemical composition is homogeneous and the molecular architecture is purely linear with no branching. Five poly-G samples were created, where the number of G monomers per chain varied from N=2 to N=32, and the numbers of chains were M=3144,1560,776,392, and 200 accordingly.

The classical CHARMM force fields [16] for molecular dynamics were used in the atomistic simulations of lignin. The potential energy function in CHARMM includes terms describing the individual covalent bonds, the angle-dependent bending of 2 consecutive bonds, the torsion of the dihedral angle of 3 consecutive bonds, and the improper out-of-plane bending of 3 consecutive bonds. As a result, the intra-molecular connectivity is defined. The potential energy function also includes non-bonded terms such as the pairwise interatomic Lennard-Jones (LJ) potentials and the electrostatic interactions between charges carried by the atoms. The original CHARMM force fields have been modified to allow more precise parameterization of the interaction parameters that match the quantum chemistry calculations on lignin as well as the experimental data on lignin’s structure. The parameters of the modified CHARMM force fields are provided by LigninBuilder [14]. Specifically, Vermaas et al. performed extensive quantum mechanical calculations of the H, G, and S lignin monomers and their various dimer combinations. The calculations generated data for developing comprehensive CHARMM force fields tailored for lignin. Compared to the general CHARMM force fields, a more accurate representation of the interactions and conformations of lignin monomers and dimers was achieved. See the developer’s GitHub repository for more details regarding the modified CHARMM force fields [17].

**Figure 2:**
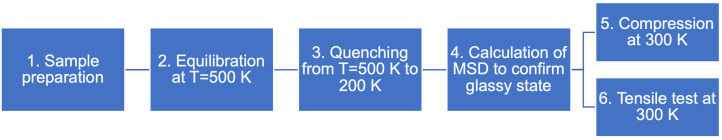
A flow chart for atomistic simulations of a lignin sample.

All the atomistic simulations were performed using Large-scale Atomic/Molecular Massively Parallel Simulator LAMMPS [18], which makes use of Message Passing Interface (MPI) for parallel communication and is a free and open-source simulation package. The cut-off radius for the pairwise LJ interaction was 12 Å, and the long-range electrostatic interactions were computed using the particle–particle particle–mesh (PPPM) solver with a precision of $10^{-4}$. The timestep for evolving the trajectory of the atomistic simulation was 1 fs. Figure 2 shows the different stages of the simulations in this work.

## 3. Results and Discussion

**Figure 3:**
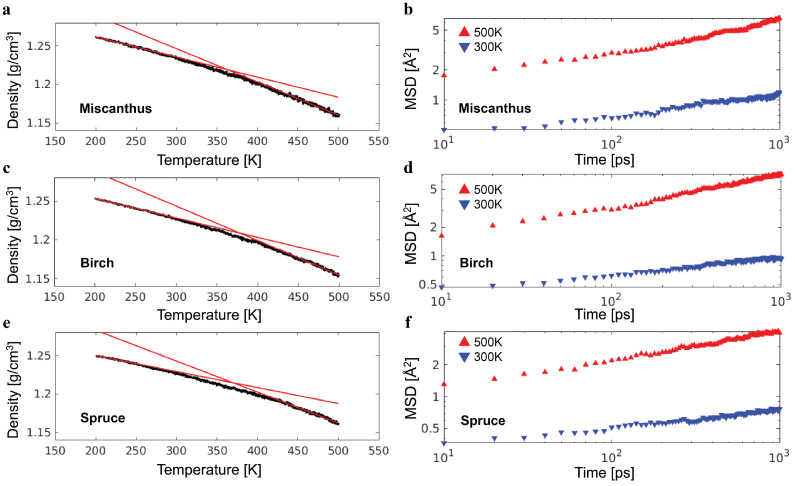
(**a**) The density of the miscanthus lignin sample as a function of temperature (black line). The two red lines indicate the fitting of high-temperature (450 K to 500 K) and low-temperature (200 K to 250 K) data to linear dependence. The intersection of the two red lines determines the glass transition temperature $T_{g}$. (**b**) The mean squared displacement (MSD) of the miscanthus lignin as a function of time at low and high temperatures. The results of the same analysis of the birch and spruce lignin samples are shown in (**c**–**f**), respectively.

The lignin molecules in a sample need to be packed in a cubic simulation box with periodic boundary conditions for the study of bulk mechanics. This was completed at an artificially high temperature of T=800 K, which sped up the uniform distribution of lignin molecules throughout the simulation box. The temperature in all simulations was controlled by a Nosé–Hoover thermostat with a characteristic damping time 50 fs. Subsequently, the sample was equilibrated at a temperature of T=500 K and pressure of P=1 atm over 0.5 million time steps. The pressure was controlled using an additional Nosé–Hoover barostat with a characteristic damping time of 500 fs. After the equilibration, the sample was quenched at P=1 atm from T=500 K to T=200 K. The quenching lasted for 3 million time steps or, equivalently, 3 ns. The glass transition temperature $T_{g}$ was determined by extrapolating the high-temperature and low-temperature simulation data of mass density and finding their intersection [19], as illustrated by the red lines in Figure 3a. It was determined that $T_{g}$ = 363 K, 379 K, and 370 K for the miscanthus, birch, and spruce lignin samples, respectively. At room temperature (T=300 K), the density was 1.23 g/cm^3^ for the three samples and comparable with the typical mass density of lignin, which is around 1.3 g/cm^3^.

To confirm a sample at T=300 K is in the glassy state, the mean squared displacement (MSD) of lignin monomers as a function of time was computed and compared with the length scale $l_{0}=\sqrt[{3}]{\left(V/N_{monomer}\right)}$ of a monomer [19], where *V* is the volume of the simulation box containing $N_{monomer}$ lignin monomers. The MSD over 1 ns, which is the time period for a unit strain at a strain rate of 10^−6^ fs^−1^, is well below the square of a lignin monomer with a size of $l_{0}^{2}$, which is around 50 Å^2^. As such, the lignin samples have rather limited molecular mobility, as expected for the glassy state, during the deformations at a strain rate of 10^−6^ fs^−1^ or higher. Discussing further details of the glassy dynamics is out of the scope of this work.

**Figure 4:**
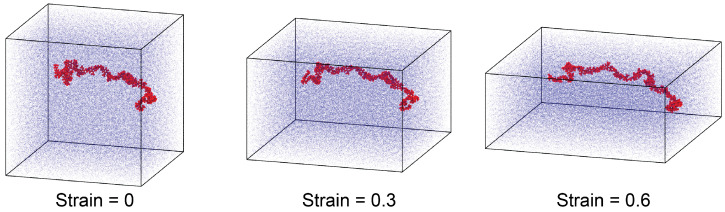
Snapshots of the poly-G lignin sample with N=32 at different compressive strains.

The glassy samples at T=300 K were used in the mechanics simulations. Two deformation modes were simulated. In one mode, uniaxial compression was applied along the *z*-direction with a true strain rate of 10^−6^ fs^−1^, and the pressure along the lateral *x*-and *y*-directions was maintained at 1 atm using a Nosé–Hoover barostat with a characteristic damping time of 500 fs. The temperature during the compression was kept at T=300 K using a Nosé–Hoover thermostat. Figure 4 visualizes the poly-G lignin sample with N=32 G monomers per chain under compressive strain, where one lignin chain is highlighted in red, while others are dimmed as the background. In the second mode, tensile loading was applied along the *z*-direction. The box expanded at a constant engineering strain rate 10^−4^ fs^−1^. Meanwhile, the box sizes in the lateral *x*- and *y*-directions were kept unchanged to introduce a tri-axial stress condition necessary for the modeling of cavitation and mechanical failure.

**Figure 5:**
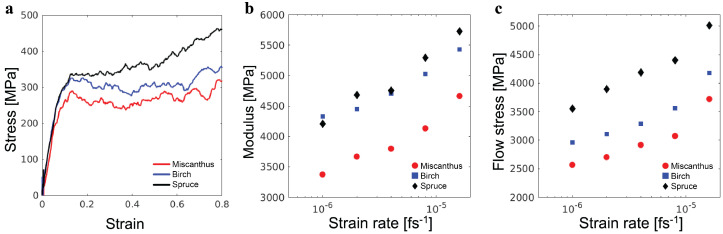
(**a**) Stress–strain curves of three representative lignin samples under compression. Elastic modulus *E* and plastic flow stress $\sigma_{flow}$ of samples under various strain rates are shown in (**b**,**c**), respectively.

The stress–strain curves for the three lignin samples under uniaxial compression are shown in Figure 5a. All curves exhibit an initial linear elastic regime, a yield peak, and then a plastic flow with ultimate strain hardening as the strain increases. These features resemble the mechanical response of amorphous glassy polymers under uniaxial compression [19]. From the initial linear elastic regime, Young’s modulus *E* is determined. The stress–strain curve at strains below 5% is fit to a linear function, with the slope being *E*. The fitting results are E=3.3 GPa, 4.3 GPa, and 4.2 GPa, for the miscanthus, birch, and spruce lignin samples, respectively. All values fall in the range of E≈3.1 GPa–6.7 GPa from the experiments on real lignin samples [20]. The modulus is comparable to that of synthetic thermoplastics such as polystyrene but higher than that of synthetic elastomers by more than one order of magnitude. This result is consistent with the existence of lignin samples as rigid powders on a macroscopic scale.

The plastic flow stress $\sigma_{flow}$ in the post-yield and pre-strain-hardening regime is determined as the average stress for 0.4<ϵ<0.5. $\sigma_{flow}=2.5$ GPa, 2.9 GPa, and 3.5 GPa for the miscanthus, birch, and spruce lignin samples, respectively. As shown in Figure 5b,c, additional simulations at higher deformation rates demonstrate that both *E* and $\sigma_{flow}$ are in the regime, where they show only weak logarithmic dependencies on the strain rate, as expected for the glassy state [21].

**Figure 6:**
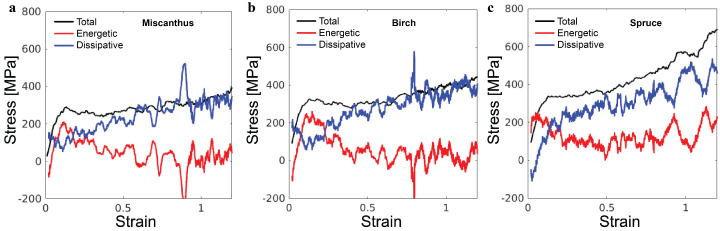
Decomposition of the total stress σ into the energetic component $\sigma_{e}$ and the dissipative component $\sigma_{d}$ as a function of the compressive strain for (**a**) miscanthus, (**b**) birch, and (**c**) spruce lignin samples.

The simulations enable the decomposition of the overall stress σ to an energetic component $\sigma_{e}$ and a dissipative component $\sigma_{d}$. This decomposition follows the first law of thermodynamics, which states that work *w* and heat transaction *q* are two equivalent ways of increasing the internal energy *u*. From δu=w+q, the heat dissipated out of the system is −q=w−δu, and therefore, $\sigma_{d}=\sigma -\sigma_{e}$ [22]. In the simulations, the dissipation of heat is realized via the thermostat. The black lines in Figure 6 show the total stress σ for the three lignin samples. The red lines are the corresponding $\sigma_{e}$, which is computed as the gradient of internal energy density with respect to strain. The blue lines are the corresponding $\sigma_{d}$. In all cases, while $\sigma_{e}$ contributes significantly to the initial elastic regime and the yielding, $\sigma_{d}$ is the major component for the stress rise in the strain-hardening regime. Such decomposition of the overall stress closely matches the behavior of synthetic polymer glasses in coarse-grained molecular simulations, demonstrating the manifestation of the generic glassy polymer mechanics in the lignin samples [19]. As such, our extensive knowledge of glassy polymer mechanics may be used to guide tailoring of the mechanics of lignin-based materials.

**Figure 7:**
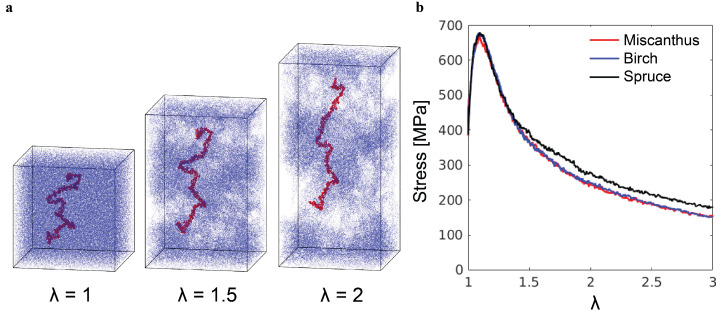
(**a**) Snapshots of the poly-G lignin sample with N=32 at different stretching ratios λ during the tensile test. (**b**) Tensile stress–λ curves of three representative lignin samples.

The tensile loading results in a qualitatively different mechanical response, as shown in Figure 7. After the initial linear elastic regime and the yield peak, the tensile stress decreases with increasing stretch $\lambda =L_{z}/L_{z}^{0}$, where $L_{z}^{0}$ and $L_{z}$ are the initial and current box sizes in the *z*-direction. The stress drop is a consequence of cavitation and catastrophic chain pullout, as illustrated by the snapshots in Figure 7a. The stretch in the simulations is the local stretch of lignin molecules, rather than the stretch of a lignin sample in experiments. On the macroscopic scale, a lignin sample exists in the powder form and does not possess any measurable stretchability. Such brittleness is related to the microscopic failure mechanism revealed by the simulations here.

**Figure 8:**
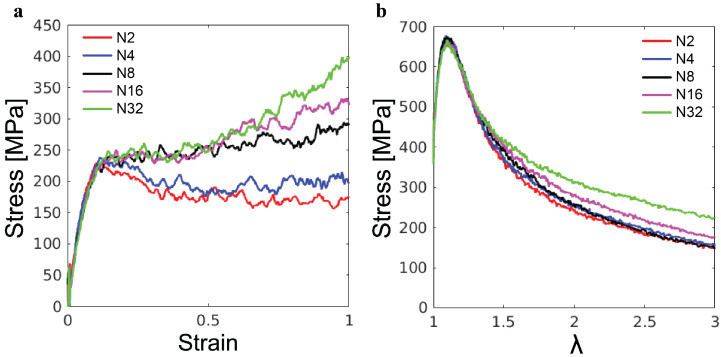
(**a**) Compression stress–strain curves and (**b**) tensile stress–λ curves of poly-G lignin samples with indicated degree of polymerization.

The simulations of the poly-G samples with varying chain lengths *N* further reveal the effects of molecular weight on the mechanical response of lignin. As shown in Figure 8a, for the uniaxial compression, the initial elastic and yield stress both exhibit almost no dependence on *N*. By contrast, *N*-dependencies are observed in the post-yield regime. For N=2 and N=4, there is a slight drop in stress following the yield peak, indicating strain softening, but eventually, the stress stabilizes in the steady state. For N>8, there is no strain softening and the plastic flow is almost independent of *N* for compressive strain up to 0.5. However, the ultimate strain hardening becomes stronger as *N* increases. Based on our understanding of strain hardening in synthetic polymer glasses, the strain hardening for larger *N* is stronger because longer molecules need more plastic rearrangements of their conformations to accommodate the same global strain [19]. The different molecular weights of the lignin samples in Figure 8a may explain their different strain-hardening behaviors. The enhancement of strain hardening from miscanthus to birch and then to spruce is related to the increase in the average molecular weight from 2.3 kDa to 4.4 kDa and then to 23.6 kDa (see Table 1).

The tensile stress–stretch curves for the poly-G samples are shown in Figure 8b. As in the compression test, there is almost no *N*-dependence of the initial elastic response and yielding. The post-yield tensile stress for larger local stretching of molecules increases with *N*, as it is harder to separate longer poly-G chains from each other. For synthetic polymer glass, it has been shown that the molecular weight has to be sufficiently high to allow the formation of entanglements and thus arrest the catastrophic chain pullout [21,23]. Clearly, up to N=32, the poly-G chains are not sufficiently long for the emergence of any entanglements. Comparing Figure 8b and Figure 7b, one may explain the higher tensile stress of the spruce lignin sample at large λ as the result of its higher molecular weight compared to the other lignin samples. For the mechanics under both compression and tension, the mechanics of lignin samples are anticipated to be affected by their chemical composition and branching coefficients as well, which needs further study in the future to clarify.

## 4. Conclusions

This work shows that the standard protocols in the molecular simulation of synthetic polymer glass mechanics may be employed to study the mechanics of lignin samples. Based on the modified CHARMM force fields and the molecular information provided for miscanthus, birch, and spruce lignin in LigninBuilder, the atomistic simulations properly capture the mechanical properties of lignin. The simulations reproduce the rigidity and brittleness of lignin, in consistency with the existence of lignin as a rigid powder at room temperature. Further analysis provides microscopic insights into the rigidity and brittleness, with knowledge borrowed from the mechanics of synthetic thermoplastics. The atomistic simulations of poly-G samples with varying chain lengths examine the effects of molecular weight, suggesting that the differences in the mechanics of the three lignin samples may be partly due to their differing molecular weights. It is anticipated that the in silico microscope established here may be extended to investigate the mechanical behavior of lignin-based materials such as adhesives, coatings, composites, and carbon fibers and thus aid in the valorization of lignin in the context of material mechanics and mechanical engineering.

## Figures and Tables

**Table 1:** Properties of three representative lignin samples from LigninBuilder [14].

Properties	Miscanthus	Birch	Spruce
Number of G monomers	282	471	3343
Number of H monomers	24	0	175
Number of S monomers	306	468	36
$M_{n}$ (kDa)	1.24	1.88	6.44
$M_{w}$ (kDa)	2.32	4.45	23.63
Branching coefficient	0	0.055	0.301

## Data Availability

The data presented in this study are available on request from the corresponding author. The data are not publicly available due to the large size.
